# Supplementary material for: Tead1a Initiates Transcriptional Priming Through the TEAD1a/YAP‐Notch1‐Spi1/Cebpα Axis to Promote Neutrophil Fate
Source: Adv Sci (Weinh). 2025 Aug 28;12(41):e05441. doi: 10.1002/advs.202505441 (PMC12591127; doi:10.1002/advs.202505441)
Supplement: Supplementary file 4 — Supplementary Dataset [file ADVS-12-e05441-s004.zip › 3-5.zip!/3-5/02-5.cluster/cluster13/Enrich/ego_BP.pdf]

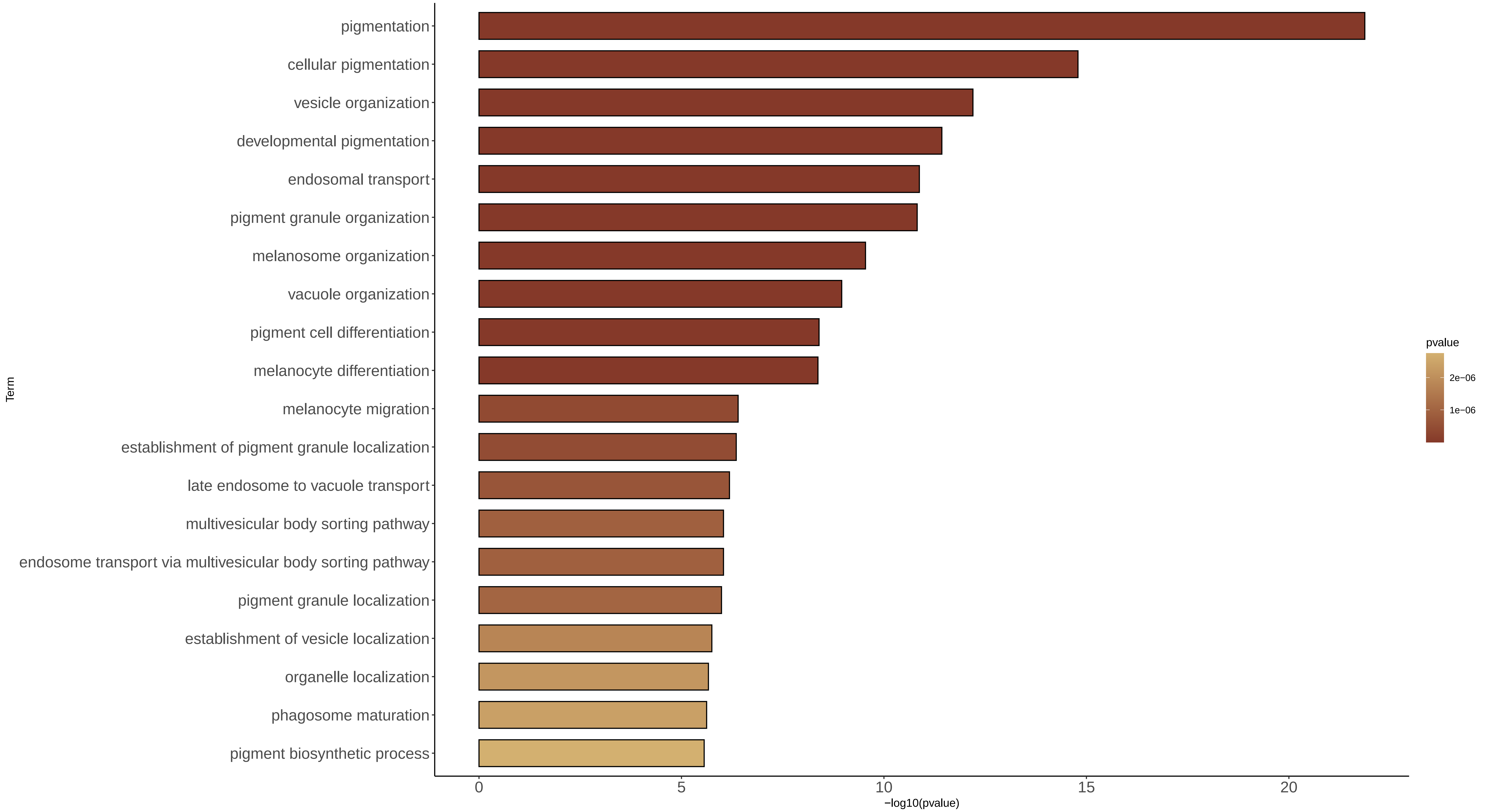
pigmentation
cellular pigmentation
vesicle organization
developmental pigmentation
endosomal transport
pigment granule organization
melanosome organization
vacuole organization
pigment cell differentiation
melanocyte differentiation
melanocyte migration
establishment of pigment granule localization
late endosome to vacuole transport
multivesicular body sorting pathway
endosome transport via multivesicular body sorting pathway
pigment granule localization
establishment of vesicle localization
organelle localization
phagosome maturation
pigment biosynthetic process
Term
0
5
10
15
20
−log10(pvalue)
pvalue
2e−06
1e−06
